# Boxwood blight: an ongoing threat to ornamental and native boxwood

**DOI:** 10.1007/s00253-018-8936-2

**Published:** 2018-04-02

**Authors:** Nicholas LeBlanc, Catalina Salgado-Salazar, Jo Anne Crouch

**Affiliations:** 1 Mycology and Nematology Genetic Diversity and Biology Laboratory, United States Department of Agriculture, Agricultural Research Service, 10300 Baltimore Avenue, Beltsville, MD USA; 2 Oak Ridge Institute for Science and Education, ARS Research Participation Program, Oak Ridge, TN USA

**Keywords:** *Calonectria*, Boxwood, Emerging pathogen, *Buxaceae*

## Abstract

Boxwood blight is an emerging disease of ornamental and native boxwood plants in the family *Buxaceae*. First documented in the 1990s at a single location in England, the disease is now reported throughout Europe, Asia, New Zealand, and North America. To address the growing concern over boxwood blight, ongoing research focuses on multiple biological and genetic aspects of the causal pathogens and susceptible host plants. Characterization of genetic variation among the *Calonectria* fungi that cause boxwood blight shows that two unique sister species with different geographic distributions incite the disease. Studies of the pathogen life cycle show the formation of long-lived survival structures and that host infection is dependent on inoculum density, temperature, and humidity. Host range investigations detail high levels of susceptibility among boxwood as well as the potential for asymptomatic boxwood infection and for other plants in the family *Buxaceae* to serve as additional hosts. Multiple DNA-based diagnostic assays are available, ranging from probe-based quantitative PCR assays to the use of comparative genomics to develop robust diagnostic markers or provide whole genome-scale identifications. Though many questions remain, the research that continues to address boxwood blight demonstrates the importance of applying a multidisciplinary approach to understand and control emerging plant diseases.

## Introduction

Boxwood blight disease (also known as box blight or buxus blight) is a significant concern for the ornamental horticulture industry and is a growing threat to established landscapes and native ecosystems alike. Not only has this new disease already been found on multiple continents, but the most susceptible host is also the most widely grown as a woody ornamental plant. Two previously unknown species of fungi have been shown to cause the disease. This combination of novel pathogens and widely grown, susceptible hosts presents enormous challenges for disease control, the production of the hosts in the nursery trade, and regulation intended to mitigate the spread of boxwood blight.

Plants susceptible to boxwood blight are members of the family *Buxaceae*, with the primary economic hosts commonly referred to as boxwood or box, in the genus *Buxus*. Boxwood have a long history of cultivation and are often a principal woody plant in built landscapes and historic gardens (Batdorf 2004). In many parts of the world, non-cultivated, indigenous boxwood are also common components of native ecosystems, with multiple species listed as endangered (Batdorf 2004; Domenico et al. 2012; IUCN Red List of Threatened Species 2017).

Boxwood also have significant economic value. In the USA, for example, these plants represent the greatest proportion (approx. 15%) of sales among broadleaf evergreens, with an estimated total annual value of 126 million US dollars (USDA-National Agricultural Statistics Service Census of Agriculture 2014 reports, https://www.agcensus.usda.gov). Among the different cultivars of boxwood, *Buxus sempervirens* ‘Suffruticosa’ (English boxwood) is one of the most popular and commonly grown types and is also among the most susceptible to boxwood blight. However, as a direct result of the disease, in parts of the world where boxwood blight is present, English boxwood is now rarely sold in the nursery trade.

The purpose of this mini-review is to provide an overview of recent research advances focused on boxwood blight. As mitigation strategies and chemical control of boxwood blight were the primary focus of a recent review (Palmer and Shishkoff 2014), these subjects will not be covered. Instead, the first part of this review centers on the historical emergence of the disease and the coincident description of the two causal pathogens. This material is followed by a summary of the pathogen life cycle, a discussion of variation in host susceptibility, and a recounting of recent efforts to develop diagnostic assays for pathogen detection. Finally, potential future research is discussed. Overall, this review highlights the significant ongoing contributions by the diverse international research groups that are working to understand and manage the boxwood blight pathosystem.

## Emergence of boxwood blight on ornamental and native boxwood

**Fig. 1:**
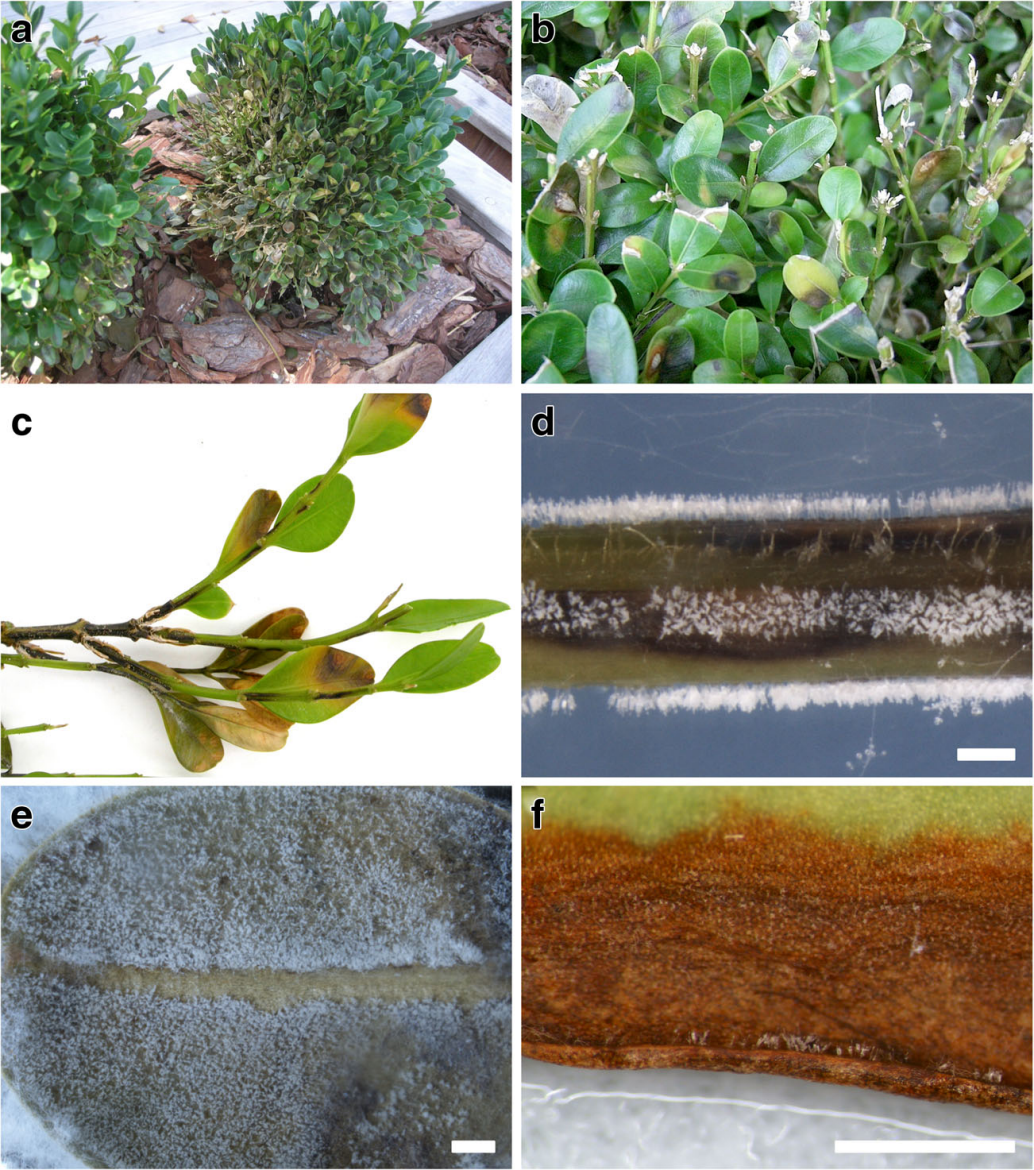
Boxwood blight symptoms and pathogen signs. **a**, **b** Disease symptoms on boxwood in the landscape, including brown leaf spots and defoliation. **c** Close-up view of typical circular brown leaf spots and black streaking on stem tissue (**a**–**c** pictures courtesy of M. Daughtrey). **d** Sporulation of the pathogen on stems along black streaks. **e**, **f** Sporulation on infected leaves after incubation in wet chamber. Scale bars: **d**–**f** = 500 μm

In 1994, a new blight disease was found on boxwood from a single nursery in southern England (Henricot and Culham 2002; Henricot 2006). Refer to Fig. 1a–c for images of characteristic disease symptoms and Fig. 1d–f for characteristic signs of the pathogen. By the late 1990s, similar disease symptoms were found on boxwood from multiple locations in England, in surrounding countries of the United Kingdom, and in New Zealand (Crous et al. 2002; Henricot et al. 2000; Henricot and Culham 2002; Henricot 2006). Subsequent reports of boxwood blight document the progressive spread of the disease across Europe and into Asia over a period of 15 years. Boxwood blight appeared in Germany in 2005, Belgium and France in 2006, Spain and Italy in 2008, Croatia in 2009, and the Czech Republic in 2010 (Brand 2005; Cech et al. 2010; Crepel and Inghelbrecht 2003; Pintos Varela et al. 2009; Saracchi et al. 2008; Šafránková et al. 2012; Saurat et al. 2012). The disease was first identified in Asia in the Republic of Georgia in 2010 and Abkhazia in 2011, where it affected native stands of *B. colchica* (Gasich et al. 2013; Gorgiladze et al. 2011). Since then, boxwood blight has been found throughout the native forests of Iran and on wild native *B. sempervirens* in Turkey where up to 90% of some boxwood populations were completely defoliated just 1 year after the first detection in 2011 (Akilli et al. 2012; Lehtijärvi et al. 2014, 2017; Mirabolfathy et al. 2013).

Boxwood blight was first found on the North American continent in the fall of 2011. Initial reports were from the east coast of the USA (Ivors et al. 2012) and the west coast of Canada (Elmhirst et al. 2013). Since then, the disease has been found at multiple locations across the eastern USA, including four states in the mid-Atlantic region and the southern state of Kentucky (Malapi-Wight et al. 2014a; Ward Gauthier et al. 2016). More recently, boxwood blight was reported from plants originating from a nursery in Oregon on the west coast of the USA, but grown in the southeastern state of Florida (Iriarte et al. 2016). Altogether, at the time of this writing, boxwood blight has been reported from 25 states (LaMondia and Shishkoff 2017; Williams-Woodward 2015).

The increasing number of boxwood blight outbreaks across the European continent and the USA suggests that the pathogen may have been spread via anthropogenic pathways, such as inadvertent transport of infected nursery stock. However, reports of the disease in native ecosystems and a lack of information surrounding the geographic origins of the fungi that cause the disease raise unanswered questions (Akilli et al. 2012; Gasich et al. 2013; Gorgiladze et al. 2011; Lehtijärvi et al. 2014, 2017; Mirabolfathy et al. 2013). For example, are all of these outbreaks due to human-mediated movement of the pathogen? Ongoing research on the genetic variation of the causal pathogens of boxwood blight will likely refine our understanding of the genetic variation, movement, and evolution of these organisms.

## Genetic variation and reproduction of the fungi causing boxwood blight

Two sister species of fungi in the genus *Calonectria* cause boxwood blight disease. Due to taxonomic revisions, multiple names may be found associated with these pathogens; however, the currently accepted names are *Calonectria pseudonaviculata* (Crous, J.Z. Groenew. & C.F. Hill) L. Lombard, M.J. Wingf. & Crous (syn. = *Cylindrocladium buxicola* Henricot; *Cy. pseudonaviculatum* Crous, J Z. Groenew. and C. F. Hill) and *C. henricotiae* Gehesquiére, Heungens and J.A. Crouch (Gehesquière et al. 2016). The first phylogenetic survey of boxwood blight fungi identified the pathogen as a member of the asexual genus *Cylindrocladium* (syn. = *Calonectria*), but suggested that the pathogen was a novel species (Henricot et al. 2000). Subsequent multi-locus sequencing and phylogenetic analysis of isolates from the United Kingdom and New Zealand further supported the pathogen’s identification as a novel *Cylindrocladium* (=*Calonectria*; Lombard et al. 2010) species (Crous et al. 2002; Henricot and Culham 2002)*.* Two competing names were proposed for the pathogen during 2002: *Cy. pseudonaviculatum* and *Cy. buxicola*. However, since *Cy. pseudonaviculatum* was published several months earlier, this stood as the accepted species name, despite proposals to use *Cy. buxicola* due to widespread usage in Europe (Henricot et al. 2012; May 2017). Measurement of amplified fragment length polymorphisms (AFLPs) among a wide collection of *C. pseudonaviculata* isolates from these same two countries found little intraspecific genetic variation (Henricot and Culham 2002).

A second fungal species causing boxwood blight was described 14 years after the first species based on phylogenetic analyses of a collection of 28 pathogen isolates from the UK, Europe, and the USA (Gehesquière et al. 2016). DNA sequence analysis from four nuclear markers identified two well-supported sister clades. One clade contained 16 isolates and included the type specimen of *C. pseudonaviculata*. The remaining 12 isolates were named as members of a new pathogen species, *C. henricotiae* (Gehesquière et al. 2016). Consistent with the previous observation of minimal genetic variation, neither of the two species exhibited intraspecific variation across the sequenced loci (Gehesquière et al. 2016). To date, *C. pseudonaviculata* has been found in every country that has reported boxwood blight, while *C. henricotiae* has only been reported in four countries in continental Europe and the UK (Gehesquière et al. 2016).

Studies assessing sexual reproduction suggest that *C. henricotiae* and *C. pseudonaviculata* are not self-compatible (i.e., homothallic) and generally do not undergo sexual reproduction, but the current data are inconclusive on this point. Initial pairwise mating combinations of *C. pseudonaviculata* isolates failed to produce evidence of sexual recombination (Henricot and Culham 2002). Similarly, pairing isolates between and within the two species did not yield any signs of mating (Gehesquière et al. 2016). More recently, use of comparative genomics showed that the boxwood blight pathogens are heterothallic, as defined by the presence of just a single mating-type gene per isolate genome (Malapi-Wight et al. 2014b). Interestingly, from a sample of 237 *C. pseudonaviculata* isolates and 31 *C. henricotiae* isolates, all isolates of *C. henricotiae* were of the *MAT1-1* mating-type, while all isolates of *C. pseudonaviculata* were *MAT1-2* (Malapi-Wight et al. 2014b). Based on the known distribution of the two species, this could mean that in North America, Asia, and parts of Europe where only one species resides, mating potential is limited due to the presence of just a single mating type. Barren perithecia were produced from interspecific laboratory pairings of *C. henricotiae* and *C. pseudonaviculata*; however, these structures were also observed when fungi were paired with themselves (Crouch, personal communication). This shows that the mating-type determinants of these fungi do not impede early stages of the sexual cycle. Because perithecia were barren, it is still unknown if the sexual cycle can be completed. Light source and culture medium both play a role in perithecium production, with a 12-h photoperiod conducive to, and total darkness unfavorable for, fruiting body development. It is likely that further optimization of environmental conditions will be required to address unanswered questions in this area (Crouch, personal communication). Altogether, these studies have shown that there is no apparent role for sexual reproduction among or between populations of *C. pseudonaviculata* and *C. henricotiae*.

Several whole genome sequence assemblies with varying levels of completion are available for both *C. henricotiae* and *C. pseudonaviculata*, curated together at a single website (Crouch et al. 2017). The 55.0 Mb genome assembly of a North American isolate CBS 139707 (also known as cpsCT1) is assembled into just 27 scaffolds and is predicted to contain 16,304 genes (Crouch et al. 2017). These genome sequences, along with assemblies for related fungi *C. leucothoes*, *C. naviculata*, and *C. pseudoreteaudii*, have already been employed for studies of mating-type, diagnostic marker development, and whole genome-scale sequence comparison of isolates from different hosts (Malapi-Wight et al. 2014b, 2016a, b; Ye et al. 2017). Moving forward, these resources are likely to yield additional information about the genetic diversity of the boxwood blight pathogens.

## Life cycle of the boxwood blight fungi

The life cycle of the fungi causing boxwood blight has been studied primarily in the context of how the abiotic environment influences pathogen fitness. To date, research on the life cycle of the boxwood blight pathogens suggests that variation in climatic conditions plays a major role in disease epidemiology. Similar to other pathogens in the genus *Calonectria*, the fungi that cause boxwood blight can initiate infection from asexual conidia and also form long-lived survival structures called microsclerotia. The life cycle of *C. pseudonaviculata* is better studied than that of *C. henricotiae*, primarily because *C. pseudonaviculata* has been known since the late 1990s, whereas *C. henricotiae* was only formally recognized in 2016. Furthermore, in regions of the world where *C. henricotiae* is not present in the environment, experiments with this species must be performed under containment conditions (e.g., LaMondia and Shishkoff 2017) and field studies are prohibited. It is not unreasonable to assume that *C. henricotiae* displays a similar infection strategy to that of *C. pseudonaviculata*, but this remains untested. However, some phenotypic differences have been observed between the two species. In particular, *C. henricotiae* is more tolerant of heat and antifungal compounds (Gehesquière et al. 2016; Shishkoff 2016), whereas *C. pseudonaviculata* shows greater tolerance toward sanitizing agents such as ZeroTol 2.0 (Shishkoff 2016). Future work will need to resolve how these phenotypic differences translate into variation in the epidemiology of boxwood blight caused by these two species. In this section, unless otherwise specified, the information presented refers to work with *C. pseudonaviculata*.

**Fig. 2:**
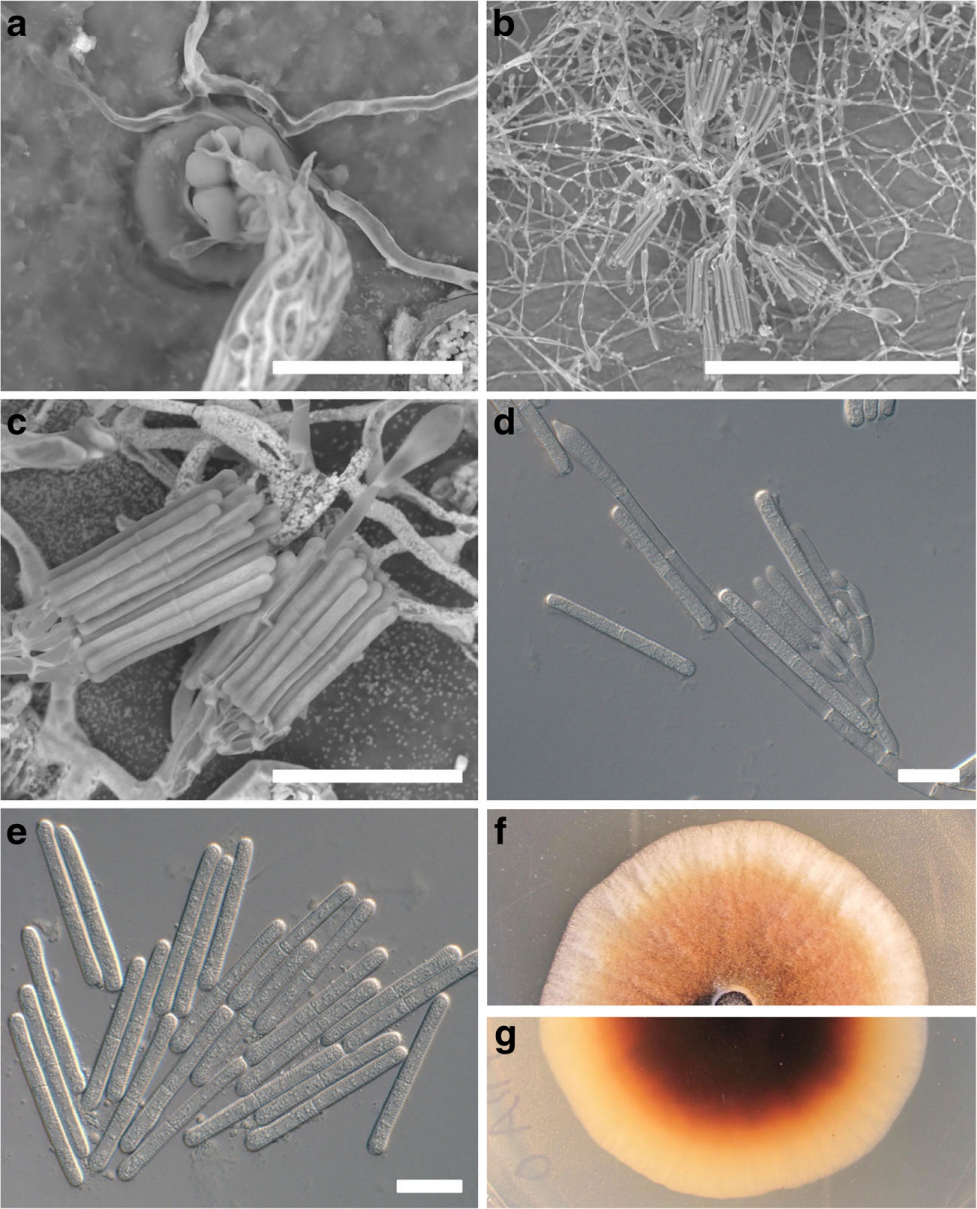
Microscopic observations of the pathogen on infected boxwood leaves and on culture media. **a** A scanning electron microscope (SEM) image of *Calonectria pseudonaviculata* penetrating a leaf stoma. **b**, **c** SEM images of *C. pseudonaviculata* sporulation on leaf tissue, including conidiophores, conidia, and vesicles. **d**, **e** Conidiophores, conidia, and a vesicle produced by *C. pseudonaviculata* growing on potato dextrose agar (PDA). **f**, **g** Morphological characteristics of *C. pseudonaviculata* growing on PDA (**f** is top of culture; **g** is bottom of culture). Scale bars: **a** = 30 μm, **b** = 200 μm, **c** = 50 μm, **d**, **e** = 20 μm

*Calonectria pseudonaviculata* infects susceptible hosts via stomata on the abaxial leaf surface or directly through the cuticle, sometimes facilitated by appressorial infection structures (Fig. 2a; Henricot et al. 2008; LaMondia and Shishkoff 2017). Infection through the upper leaf surface is possible, although symptoms are reduced, possibly due to the reduction in the number of stomata on the adaxial surface (Guo et al. 2015; LaMondia and Shishkoff 2017; Shishkoff et al. 2015). Conidia are the main source of inoculum. They are produced either as primary inoculum from melanized resting structures called microsclerotia, or as secondary inoculum emerging from boxwood leaves or twigs (Gehesquière et al. 2013). Conidial dissemination is thought to occur mainly through water splash, or mechanical transfer via contaminated tools, animals, or other means (Gehesquière et al. 2013; Henricot 2006). Windborne dispersal of conidia has been shown to be extremely rare (Gehesquière et al. 2013). The lack of windborne dispersal may be due to physical limitations, as the conidia of both *C. henricotiae* and *C. pseudonaviculata* are relatively large and are contained within a thick liquid substance (Fig. 2b–e; Gehesquière et al. 2013, 2016; Henricot and Culham 2002).

Inoculum density is a key factor for disease development, with higher numbers of conidia leading to greater disease incidence across a tested range of 1250 to 40000 conidia/mL on 2-year-old plants (Avenot et al. 2017). Less susceptible cultivars such as *Buxus* x ‘Green Mound’, *B. sinica* var. *insularis* ‘Nana’, and *B. microphylla* ‘John Baldwin’ show no symptom development at inoculum doses below 5000 spores/mL, but it is not known whether latent infection occurred under these conditions (Avenot et al. 2017). Germination of conidia is increased when they are exposed to 24 h of darkness following a 14-h photoperiod, compared to germination rates when conidia are exposed to 12 or 24 h of light following the same 14-h photoperiod (Marine et al. 2017). This is consistent with findings that show numerical—but non-significant—increases in disease severity on shoots of susceptible cultivars *B. sempervirens* ‘Suffruticosa’ and *B. sempervirens* ‘Justin Brouwers’ exposed to 12 or 6 h of dark after inoculation by *C. pseudonaviculata* conidia (Marine et al. 2017). Together, these findings illustrate the need to either prune out infested branches or completely remove diseased plants from the nursery or landscape, to reduce the potential inoculum load in the environment, and to increase light penetration into the canopy to reduce the pathogen’s ability to initiate fresh infections.

Conidia germinate 3 h after inoculation, and penetration occurs 5 h post-inoculation (Henricot et al. 2008). Five days after infection, the pathogen can be seen re-emerging from the abaxial leaf surface, and visible sporulation on leaves is evident at 7 days post-infection, sometimes resulting in many thousands of conidia on a single leaf (Fig. 1e). Following infection, the pathogen also develops clusters of melanized cells in foliar and root tissue that produce microsclerotia (Dart et al. 2015; Henricot and Culham 2002; Weeda and Dart 2012). Based on the demonstrated ability of microsclerotia to remain viable for as long as 40 weeks buried in soil, 30 months in buried leaves and stems, these structures likely serve as the key mechanism for the pathogen to overwinter and re-initiate infection of susceptible hosts (Dart et al. 2015; Shishkoff and Camp 2016). Conidia appear to have limited survival ability in the soil, although viability is observed for up to 3 weeks (Dart et al. 2015).

Variation in the survival of *C. pseudonaviculata* microsclerotia has been documented under different adverse environmental conditions. Yang and Hong (2018a) showed that younger, smaller microsclerotia are better able to survive than older, larger microsclerotia under temperature extremes ranging from − 10 to 40 °C. Microsclerotia size also affects survival of the fungus when exposed to the biocide sanitizing agent ZeroTol 2.0 (hydrogen dioxide 27.1%; Shishkoff 2016). After exposing microsclerotia to ZeroTol for 5 to 15 min, > 75% germination was still observed from large- and medium-sized *C. pseudonaviculata* microsclerotia (177–353 μm), versus only 30–50% germination of small-sized microsclerotia (125 to 177 μm) (Shishkoff 2016). Treatment of infested boxwood tissue or microsclerotia at − 10 or 30 °C kills the pathogen after 1 to 5 months (Shishkoff and Camp 2016). At temperatures within these two extremes, exposure of the pathogen to higher moisture results in greater pathogen survival rates (Shishkoff and Camp 2016). Together, these findings support the idea that by manipulating the microsclerotial environment, either through direct removal of infested plant parts or leaf litter, or by introducing conditions that induce or accelerate microsclerotial mortality, pathogen survival and levels of primary inoculum could be reduced.

Both moisture and temperature significantly influence symptom development and the fitness of the boxwood blight pathogens (Avenot et al. 2017; Gehesquière 2014; Henricot 2006). A significant reduction of disease symptoms is observed at 18 and 27 °C and disease symptoms do not develop at 29 °C (Avenot et al. 2017). Humidity also plays a key role in the infection process and pathogen growth and survival. When tested in vitro, mycelial growth, as measured by colony size, was increased at 65% RH compared to 95% RH, but it is unknown from these experiments whether any coincident changes in conidial production and/or microsclerotium formation occurred (Marine et al. 2017). In a separate study, Avenot et al. (2017) showed that by interrupting wet conditions (100% RH) for 3 h or more with a dry period (RH < 65%) at 8 h post-inoculation, a significant reduction of disease symptoms occurs. Relative to a 20-h continuous wetness period, 11× less disease was observed from this treatment (Avenot et al. 2017). These findings suggest that modification of the plant canopy to reduce humidity and increase air circulation may be a productive strategy to reduce boxwood blight, as is recommended for several plant diseases (Tivoli et al. 2013).

## Additional hosts, variation in boxwood susceptibility, and prospects for resistance breeding

The long-lived microsclerotia produced by the boxwood blight fungi are just one of the hurdles that must be overcome before this disease can be effectively controlled. Variation in susceptibility among and within different species of boxwood presents one of the greatest challenges for identifying sources of resistance to the disease. This is compounded by the fact that different levels of susceptibility have been reported for the same cultivars when assessed in different studies. There are several possible factors likely contributing to these discrepancies, and ultimately, it may take some level of standardization between laboratories with respect to experimental design, host identification, pathogen genotype(s), environmental parameters, and cultivar selection to strengthen overall research findings. For example, although the genus *Buxus* comprises 95 to 100 species, the validation of basic information regarding naming, origins, ploidy, and genetic relationships among species and cultivars based on well-defined reference taxa has only recently been studied using molecular tools (Batdorf 2004; Thammina et al. 2016; van Laere et al. 2011). These studies highlighted the potential for cultivar- or species-level misidentification from even highly curated boxwood collections (Thammina et al. 2016; van Laere et al. 2011). Experimental factors may vary across studies, encompassing laboratory versus field conditions, different temperatures, and different pathogen isolates (Ganci et al. 2013; Gehesquière et al. 2016; Henricot et al. 2000; LaMondia and Shishkoff 2017; Shishkoff et al. 2015). Some experiments make use of detached stems or leaves, while others employ unrooted cuttings, and/or whole plant infections (Ganci et al. 2013; Guo et al. 2016; Henricot et al. 2000, 2008; LaMondia and Shishkoff 2017; Shishkoff et al. 2015). Detached leaves and unrooted cuttings provide an inexpensive, high-throughput method to screen large numbers of boxwood for resistance; however, there are reported instances where the response of detached boxwood plant parts differed from the response of living hosts (Guo et al. 2016; LaMondia and Shishkoff 2017). LaMondia and Shishkoff (2017) pointed out the need to couple detached leaf resistance screenings with evaluations of whole plants to account for all components of resistance, including systemic resistance factors that are only triggered in living plant material and physical components such as plant architecture (Avenot et al. 2017; Orlowska et al. 2013; Tivoli et al. 2013).

Despite the observed variation in host susceptibility to boxwood blight, and differences in the conclusions drawn between the different studies performed to date, some generalizations can be made as to which cultivars and species of boxwood are among the most susceptible. In particular, the extensively grown *B. sempervirens* ‘Suffruticosa’ (English boxwood) consistently ranks among the most susceptible hosts. Many—but not all—cultivars of *B. sempervirens* also exhibit high levels of susceptibility to boxwood blight. However, with over 400 named cultivars of *B. sempervirens*, sources of resistance within the genus may exist (Thammina et al. 2016). In general, *B. microphylla* cultivars are among the least susceptible boxwood (Ganci et al. 2013; Guo et al. 2015, 2016; Henricot et al. 2000, 2008; LaMondia and Shishkoff 2017; Shishkoff et al. 2015).

The host range of *C. pseudonaviculata* extends to other plants in the *Buxaceae* family; it is not known whether *C. henricotiae* infects plants other than boxwood. Experimental inoculations of an unidentified species of *Sarcococca* with *C. pseudonaviculata* and subsequent development of blight symptoms was first reported by Henricot et al. (2008) using fungal isolates from England, but natural infections of this plant have not been reported from the UK. Subsequently, blight symptoms were identified from *Sarcococca hookeriana* (common name Himalayan sweet box) growing adjacent to boxwood plants in a Maryland landscape in the USA (Malapi-Wight et al. 2016b). Through experimental infection and whole genome sequencing, the *S. hookeriana* pathogen was confirmed as *C. pseudonaviculata* and shown to differ from an isolate of the fungus from an adjacent boxwood plant by just a single nucleotide polymorphism in a non-coding region of the genome (Malapi-Wight et al. 2016b). A subsequent report of a natural infection of *S. hookeriana* by *C. pseudonaviculata* was made from the USA state of Virginia, also in conjunction with blighted boxwood planted in the same landscape bed (Kong et al. 2017a). Infection of *Pachysandra terminalis* (common name Japanese spurge) with an isolate of *C. pseudonaviculata* recovered from a symptomatic boxwood plant was first demonstrated experimentally (LaMondia et al. 2012). Since then, *C. pseudonaviculata* has been found causing disease on *Pachysandra terminalis* and *P. procumbens* growing in the landscape (Kong et al. 2017b; LaMondia and Li 2013).

## Diagnostic assays for pathogen detection

Multiple molecular diagnostic assays have been developed for the detection and quantification of the causal pathogens of boxwood blight. All of the currently available assays are based on some application of polymerase chain reaction (PCR) or isothermal amplification technology to identify the DNA of target pathogens (Gehesquière et al. 2013, 2016; Malapi-Wight et al. 2016a). However, the approaches to develop individual assays as well as their ease of use and effectiveness differ.

The first published diagnostic assays developed for boxwood blight are based on real-time PCR detection of two nuclear locus targets: the multiple-copy rDNA internal transcribed spacer (ITS) and the single-copy β-tubulin 2 (*TUB2*) gene. Comparison of the two assays shows a trade-off between specificity and sensitivity. The ITS assay detects lower concentrations of pathogen DNA (10 fg), but false positive signals from non-target fungi are documented (Gehesquière et al. 2013). In contrast, with the SYBR-green-based *TUB2* detection assay false positives are not reported, but the assay requires 2–5 more reaction cycles for pathogen detection compared to the ITS assay. Application of these assays demonstrates their potential to detect *C. pseudonaviculata* in air, water, and plant samples. However, given the false positives of the ITS assay, reduced sensitivity of the *TUB2* assay, and the subsequent description of the second species *C. henricotiae* from Europe, these assays may have limited application (Gehesquière et al. 2016).

Two loop-mediated isothermal amplification (LAMP) assays are also available for specific detection of *C. henricotiae* and *C. pseudonaviculata* (Malapi-Wight et al. 2016a). This isothermal method of DNA amplification does not require the use of thermal cyclers for DNA amplification and is increasingly being applied for rapid diagnostics of plant pathogens (e.g., Ash et al. 2014; Sillo et al. 2017). The LAMP assays were developed by comparing the draft genome sequences of *C. pseudonaviculata* and *C. henricotiae* with the genome sequences of three non-target fungi, from which a set of candidate diagnostic loci and LAMP primers were identified. To validate the specificity of the LAMP primer sets, the authors screened them against a panel of DNA from target and non-target fungi as well as environmental DNA from boxwood plants for which the composition of fungal taxa was known (Rivera et al. 2015). Ultimately, two LAMP primer sets were identified that did not give any false positives or false negatives among all the samples. Though this work relied on laboratory-based electrophoresis for amplification detection, future application of alternative methods for amplicon visualization (e.g., Mori et al. 2001; Tomita et al. 2008) or adaptation to portable instrumentation such as OptiGene’s Genie instrument could make these assays more field accessible.

Assays to discriminate between *C. henricotiae* and *C. pseudonaviculata* are also available, providing important tools to monitor the potential spread of *C. henricotiae* into new areas. In the laboratory, cultured fungal isolates can be identified to the species level through DNA sequence analysis of the four nuclear loci originally used to discriminate the organisms or using a PCR-RFLP profile from the *TUB*2 gene (Gehesquière et al. 2016). Amplicon size assessment provides an indicator of the pathogen mating type, which can be used as indirect assessment of species identity, but needs to be confirmed through another method to take into consideration the possibility of the emergence of different *MAT1* idiomorphs across the two pathogen species (Malapi-Wight et al. 2014b). Two quantitative species-specific real-time PCR assays are also available to discriminate between *C. henricotiae* and *C. pseudonaviculata* based on histone, calmodulin, and *TUB2* DNA targets (Gehesquière et al. 2016). Species-specific detection is possible with these two assays even in the presence of the other non-target fungi (Gehesquière et al. 2016). With the ability to distinguish the two species that cause boxwood blight, these two real-time PCR assays could be applied to screen symptomatic and asymptomatic plant material in an effort to reduce the spread of the geographically constrained species *C. henricotiae*.

A soil baiting bioassay is also available to detect the presence of *C. pseudonaviculata* microsclerotia from soil, with a detection limit of one microsclerotium/g soil at 1000% field capacity after 96 h (Dart et al. 2014). Based on *B. sempervirens* ‘Arborescens’ leaf disks used to bait the fungus from soil, the assay is quantitative between 1 and 10 microsclerotia/g of soil, but detection of the pathogen is strongly influenced by soil type.

Development of the diagnostic assays described above highlights some of the challenges of working with these and other emerging plant pathogens, especially when assays are developed during early investigations at a stage when population diversity is still incompletely understood. The description of new species and discovery of genetic variation at target diagnostic sites can negate the effectiveness of early diagnostic assays. Indeed, recent work made use of whole genome scale comparisons among individual isolates to account for all possible sources of variation among isolates collected from lesions of symptomatic boxwood and sarcococca plants residing in the same landscape bed (Malapi-Wight et al. 2016b). Another approach for detection under development targets proteins produced by the pathogen, rather than nucleotide sequences (Veltri et al. 2016). Since changes to DNA sequences generally occur more rapidly than changes on the amino acid level, this approach should reduce the risk of losing assay specificity. In addition, protein-based pathogen identification tools can be translated into field deployable, user-friendly immunological diagnostic assays for detecting the causal agents of boxwood blight.

## Future research and concluding thoughts

Despite the research advances highlighted in this review, many important questions about boxwood blight remain unanswered. In particular, very little is known about the molecular mechanisms underlying the interaction between susceptible hosts in the family *Buxaceae* and fungi in the genus *Calonectria* that cause boxwood blight. Aside from transcriptome data from *Buxus sempervirens* deposited with the National Center for Biotechnology’s Sequence Read Archive from the 1000 Plant Transcriptomes project (accession ERS1829209) and genic SSR markers developed from RNA-Seq data, genetic and genomic resources are not available for plants in the family *Buxaceae* (Thammina et al. 2014). In contrast, the genomes of multiple *C. pseudonaviculata* and *C. henricotiae* isolates have been sequenced and made publicly available and could serve as a platform for identifying genomic regions undergoing positive selection and potentially individual genes linked to pathogen virulence (Badouin et al. 2017; Crouch et al. 2017; Malapi-Wight et al. 2016a, b). Identifying these genes and further functional validation would contribute to monitoring variation in virulence among pathogen populations and aid in identifying sources of host resistance (Stukenbrock and McDonald 2009; Vleeshouwers and Oliver 2014).

Another largely unknown aspect of boxwood blight is the role the host microbiome may play in determining the outcome of host-pathogen interactions. Although resistance breeding holds the greatest promise to mitigate boxwood blight in the nursery trade, it does nothing to protect plants already established in the environment. Preliminary data has shown fungi in the genus *Trichoderma*—well known as agents of biological control—reside in the boxwood rhizosphere (Rivera et al. 2015). Similarly, non-indigenous fungi in the genus *Trichoderma* and bacteria in the genus *Pseudomonas* have been shown to inhibit this pathogen in vitro and reduce disease symptoms (Kong and Hong 2017; Yang and Hong 2018b). Future work that makes use of targeted meta-barcoding methods to survey the variation in prokaryotic and eukaryotic microorganisms associated with different species and genera of susceptible hosts as well as differences among common commercial cultivars of boxwood may provide useful data towards controlling the disease in established plantings. Functional trait analyses from these microbial groups or the application of shotgun metagenomics could also provide insight into variation of functional aspects among the microbiomes of different susceptible hosts. These data could also be used to inform the development of boxwood blight resistant plants (e.g., Gopal and Gupta 2016; Mendes et al. 2018).

As a final note, boxwood blight is just one of the many diseases on woody ornamental plants that also pose a threat to native ecosystems. Similar to many other pathogens of woody plants, *C. pseudonaviculata* and *C. henricotiae* are generally thought of as alien (i.e., non-indigenous) pathogens that were potentially introduced and spread via the nursery industry (Gehesquière et al. 2016). However, there may be additional or alternative explanations for the emergence of boxwood blight in native ecosystems. While the pathogens may have been introduced into these native ecosystems by human activity, the presence of the pathogens in native ecosystems may also represent indigenous populations that recently emerged due to other anthropogenic or natural mechanisms (e.g., Ghelardini et al. 2016). Regardless of the underlying mechanisms responsible for the outbreak of boxwood blight in native ecosystems, it is clear that this disease is relevant outside of the ornamental horticulture industry. Future work will need to include input from natural resource professionals and stakeholders, building on the strong foundation of multidisciplinary research focused on mitigating the negative impacts of this emerging disease in nurseries and ornamental plantings.
